# The cresty neck score is an independent predictor of insulin dysregulation in ponies

**DOI:** 10.1371/journal.pone.0220203

**Published:** 2019-07-24

**Authors:** Danielle M. Fitzgerald, Stephen T. Anderson, Martin N. Sillence, Melody A. de Laat

**Affiliations:** 1 Earth, Environmental and Biological Sciences School, Queensland University of Technology, Brisbane, Queensland, Australia; 2 School of Biomedical Sciences, The University of Queensland, St Lucia, Queensland, Australia; Massey University, NEW ZEALAND

## Abstract

Generalized obesity, regional adiposity, hyperinsulinemia and hypertriglyceridemia are all potential indicators of equine metabolic syndrome (EMS). This study aimed to assess the relationship between morphometric measurements of body condition and metabolic hormone concentrations in ponies, with and without a neck crest or generalised obesity. Twenty-six ponies were assigned a body condition score (BCS) and cresty neck score (CNS). Height, girth, and neck measurements were taken. An oral glucose test (OGT; 0.75g dextrose/kg BW) was performed and blood samples collected prior to and 2 hours post dosing. Basal blood samples were analysed for blood glucose, serum insulin, triglyceride and leptin, and plasma HMW adiponectin concentrations. Post-prandial samples were analysed for serum insulin concentration. The ponies were grouped as having a) a normal to fleshy body status (BCS ≤7 and CNS ≤2; n = 10); b) having a high CNS, but without generalised obesity (BCS ≤7 and CNS ≥3; n = 11), or c) being obese (BCS ≥8 and CNS ≥1; n = 5). Responses to the OGT indicated that both normal and insulin-dysregulated ponies were included in the cohort. Post-prandial serum insulin was positively associated with CNS (P<0.035) and ponies with a CNS ≥ 3 had 5 times greater odds of being insulin-dysregulated. The high CNS group had a greater insulin response to the OGT than those in the normal/fleshy group (P = 0.006), whereas obese ponies did not differ from the other two groups. Basal HMW adiponectin was negatively correlated with post-prandial insulin concentrations (r = -0.5, P = 0.009), as well as being decreased in the group with a high CNS, compared to the obese group (P = 0.05). Cresty neck score was more predictive of insulin dysregulation than BCS, and this may be relevant to the diagnosis of EMS. Adiponectin may also be a measure of insulin dysregulation that is independent of body condition.

## Introduction

Equine metabolic syndrome (EMS) is a cluster of metabolic derangements, including insulin dysregulation (ID; insulin resistance and/or hyperinsulinemia), hypertriglyceridemia, obesity/regional adiposity, and laminitis [1]. The early identification of ID might enable horse owners to reduce the risk of laminitis, which is a painful foot condition associated with high morbidity. Laminitis has been identified as a primary weight-related disorder upon veterinary diagnosis [2]. However, not all cases of EMS are overweight, and a lean phenotype of the syndrome exists [3]. As such, regional adiposity is thought to be a stronger identifier of EMS and the risk of developing laminitis than generalised obesity [4–7]. No studies have yet compared obese animals with animals displaying regional adiposity and an otherwise normal body condition.

**Fig 1:**
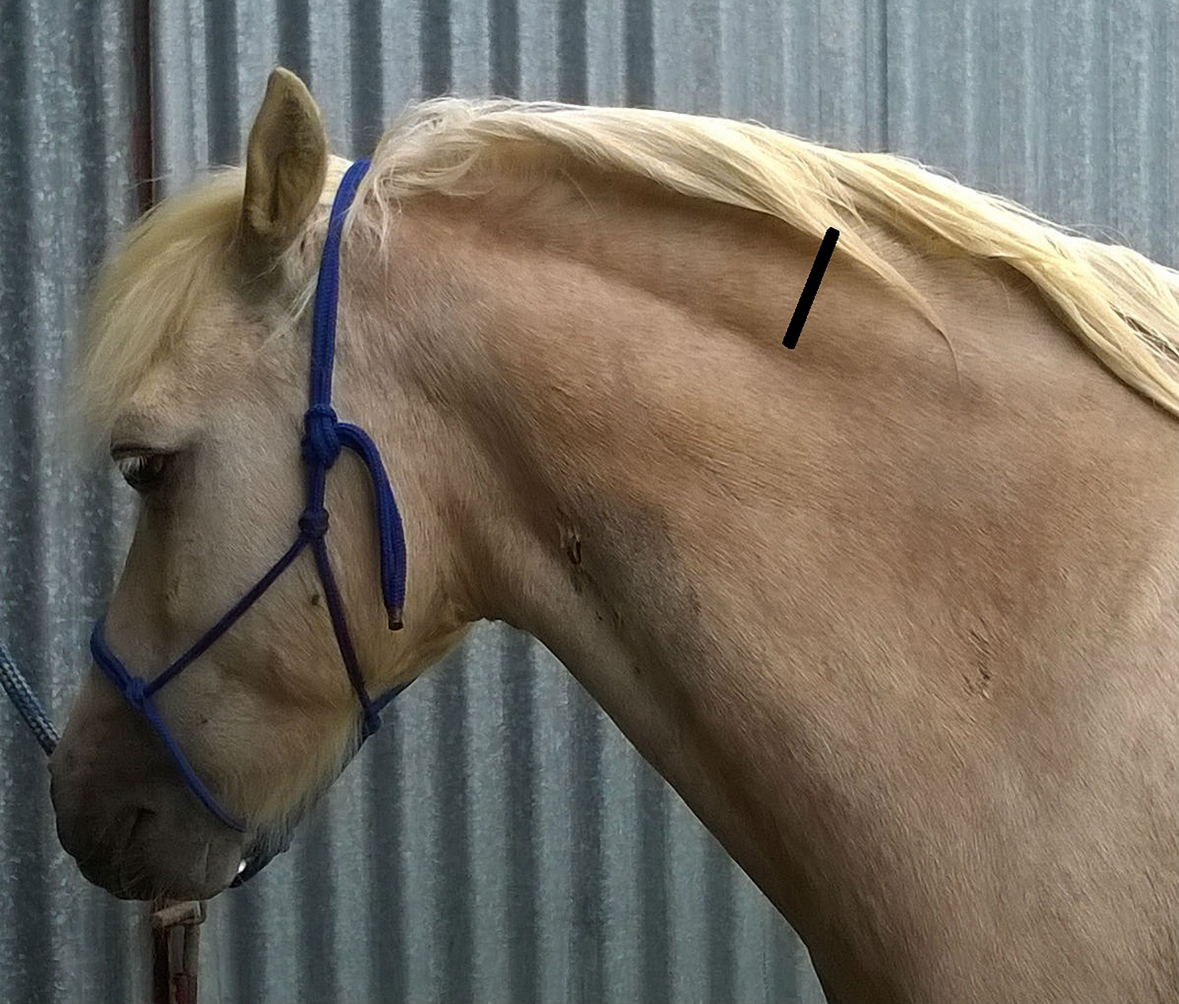
A pony with a cresty neck. A crest neck is an enlarged fat deposit along the nuchal ligament, identified by the black bar. This pony was assigned a cresty neck score of 3.

One proxy measure of body fat composition is the body condition score (BCS), where an animal is assessed against an ordinal scale of descriptive factors [8, 9]. The BCS is considered by veterinary practitioners to be a useful measure when diagnosing obesity and identifying the risk of a horse developing endocrinopathic laminitis [10]. The repeatability of BCS is good when the assessment is performed by trained practitioners, whereas horse owners have been shown to underestimate the BCS of their horses [11–13]. The cresty neck score (CNS) is a descriptive, ordinal scale used to measure nuchal fat accumulation (along the top of the neck; Fig 1), which is one site for determining regional adiposity [4]. The amount of nuchal fat can be disproportionate to the amount of total body fat and can therefore be considered as an independent measure of adiposity [9]. The CNS has been validated against nuchal fat thickness [14], where the nuchal fat was associated with total carcass fat that was measured on a 15 point scale, rather than weight on dissection.

Adipose tissue, along with being an energy store, is an active endocrine organ with roles in synthesising and secreting hormones that impact metabolism [15]. In horses, the adipokines adiponectin and leptin have been implicated in obesity, insulin resistance, and chronic laminitis [16–20]. Data from human studies have shown that visceral fat (such as omental or mesenteric fat) is associated with type 2 diabetes, insulin resistance and altered adipokine concentrations [21, 22], more so than other fat depots [23, 24]. The influence of increased adiposity on insulin regulation has been evaluated in horses [20, 25]. However, no studies have addressed whether an increased CNS alone, without generalised adiposity (i.e. increased BCS), can be used to identify ID in horses and ponies.

This study had three aims:

1. To determine if ponies with an increased CNS, but of otherwise “normal” body condition, are more likely to have ID than ponies without a lower CNS.
2. To identify whether objective morphometric measures correlate with CNS and BCS measures.
3. To elucidate whether adipokines or triglycerides were associated with morphometric measures or insulin dysregulation.

## Materials and methods

### Ethics statement

This work was carried out with approval from the Animal Ethics Committee of The University of Queensland (QUT/SVS/316/16) and Queensland University of Technology (1600000825).

### Animals

Twenty-six ponies of mixed breeds (14 males and 12 females) owned by Queensland University of Technology were evaluated visually and using palpation for BCS (assessed on a scale of 1 (very poor) to 9 (very fat) [8]), and CNS (assessed on a scale of 0 (no visual or palpable crest) to 5 (large, drooping crest), as described by [4]), by an experienced assessor. Half (n = 13) of the ponies were also assessed for BCS and CNS by a second assessor to validate the scoring technique of the first assessor (these scores were not used in any other analyses). Additional morphometric measurements were taken by the same assessor and included height (at the wither), body weight (BW), girth circumference (measured just caudal to the wither), and neck circumference (measured at the mid-point of the neck). Both girth and neck circumference are reported as a ratio of height, to correct for the variation in pony size. All assessments were undertaken prior to investigation of metabolic status.

The ponies were grouped into three exclusive body type groups. Normal to fleshy body status was designated as BCS ≤ 7 and CNS ≤ 2 and the group was labelled “Normal” given that ponies frequently have a slightly more fleshy appearance than horses [26]. Obesity was designated as BCS ≥ 8 and CNS ≥ 1 (Obese), and BCS ≤ 7 and CNS ≥ 3 was considered to be indicative of regional adiposity without generalised obesity (High CNS). No pony had a BCS < 4/9.

Each animal was evaluated for ID using post-prandial serum insulin concentrations measured during an oral glucose test (OGT). For this test, dextrose (0.75 g/kg BW; dissolved with 500 mL water) was administered in a small meal containing 200g wheat bran and 0.3% BW lucerne chaff. Insulin dysregulation was diagnosed when the post-prandial serum insulin response was ≥ 80 μIU/ml two hours after the test meal [27].

### Blood samples

Blood samples were collected via jugular venepuncture during the OGT at 0h (basal) and 2h (post-prandial). Blood glucose was measured immediately from whole blood using a hand-held glucometer previously validated [28] for equine samples (Accu-Check, Roche Diagnostics, Castle Hill, New South Wales, Australia). The remaining blood was separated into clot activator (serum) and EDTA (plasma) vacutainer tubes (Beckton Dickson, New Jersey, USA). The EDTA tubes were immediately placed in ice for 10 mins before centrifugation (1500*g* for 10 mins), and then the plasma was stored at -20°C. Clot activator tubes were left at room temperature for 30 mins before centrifugation (1500*g* for 10 mins) and stored at -20°C. Samples were then transferred to -80°C for storage within 5 days.

### Assay validation

The plasma high molecular weight (HMW) adiponectin concentration was measured using a Millipore human high molecular weight adiponectin ELISA (EZHMWAN-65K) as described by Wooldridge et al. [29]. Since the ELISA was last validated, a change to the antibody was made by the manufacturer. Therefore, the ELISA was revalidated as part of this study. Equine samples were separately pooled from ponies with expected high and low adiponectin concentrations based on their post-prandial insulin concentration [30]. Assay precision was determined by the coefficient of variation (CV) among six replicates of the pooled ‘high’ sample. Accuracy and linearity were determined by a linear dilution of both high and low pooled samples that were diluted 1:1, 3:4, 1:2 and 1:4. Additionally, the inter-assay CV was determined across two kits run on different days.

### Assays

The serum insulin concentrations were measured using a chemiluminescent assay frequently used for the analysis of equine insulin concentration [31], and the serum triglyceride concentrations were measured using enzymatic determination (AU680; Beckman Coulter, Sydney, Australia), both run at a commercial veterinary diagnostics laboratory (Vetpath, Ascot, WA, Australia). The serum leptin concentrations were measured using the Millipore Multi-Species Leptin radioimmunoassay kit (XL-85K), with results expressed as human equivalent concentrations (HE).

### Statistical analyses

All continuous variables were checked for normality of distribution (Shapiro-Wilk statistic). All outliers, identified using Grubbs test, were excluded from analysis. Continuous variables were analysed among groups using a one-way analysis of variance (ANOVA), with post-hoc pair-wise comparisons made with the Holm-Sidak method. For non-parametric data, the Kruskal-Wallis one-way ANOVA on ranks was used, with post-hoc pair-wise comparisons made with Dunn’s method. Associations between two measures were made using Pearson’s correlation coefficient. The association between the two assessor’s body composition scores was determined with Spearman’s rho statistic. The statistical program Sigma Plot v13.0 was used with a significance of P ≤ 0.05 accepted.

Body condition score and cresty neck score were included as binary conditions (BCS ≤7/9 and ≥8/9, and CNS ≥3/5 and ≤2/5) in a binary logistic regression model in the statistical program SPSS (v25) to determine the odds of a pony being insulin-dysregulated based on these two risk factors. Further binary logistic regression modelling was performed to determine the odds of a pony being insulin-dysregulated if the pony displayed both (BCS ≥8/9 and CNS ≥3/5) or one (BCS ≥8/9 and CNS ≤2/5; BCS ≤7/9 and CNS ≥3/5) of the risk factors compared to none (BCS ≤7/9 and CNS ≤2/5). The Hosmer-Lemeshow statistic was used to assess model fit, and the Nagelkerke R square value was used to assess the variability explained by the model.

## Results

### Adiponectin assay validation

The HMW adiponectin assay performed acceptably with the pooled ‘high’ sample. However, the pooled ‘low’ sample was read at the lower limit of the standard curve, and as a result an increased assay limit of detection (LOD) of 3.125ng/mL (minimum on the assay’s range or 0.625μg/mL when corrected for the dilution factor) is recommended when using this assay for equine plasma. The assay was reasonably precise, being consistent between replicates (Table 1). The recovery-on-dilution data were linear, but recovery values were consistently lower than expected (Table 1).

**Table 1:** Validation data for HMW Adiponectin ELISA assay used with equine plasma.

	Capture antibody	Standards	Precision CV, %	Accuracy, %	Linearity (r^2^)	Recovery-on-dilution %	Interassay CV, %
HMW Adiponectin	Polyclonal goat anti-adiponectin	Human HMW adiponectin					4.8
High			13.3	113	0.99	61.2	
Low			3.0	-*	0.88	-*	

Key:

*upon dilution, low samples were below the standard curve. Recommended limit of detection (LOD) for equine samples is 0.625μg/mL, HMW; high molecular weight

### Animal groups

There was significant association between assessor’s scores for both BCS (r = 0.89, P < 0.01) and CNS (r = 0.66, P = 0.01). Of the twenty-six ponies, ten were grouped as “normal” (four females and six males, 15.7 ± 6.8 y), five were “obese” (three females and two males, 10.2 ± 5 y), and eleven were classified as “high CNS” (five females and six males, 10.7 ± 4.3 y).

### Morphometric measurements

The differences in morphometric measures among groups are described in Table 2. There was no difference in height at the withers or BW among the groups (Table 2). As expected, because these variables were used to classify the ponies into groups, the BCS differed significantly among groups, and the CNS was higher in the high CNS group. The girth to height ratio was greater in the obese group compared to both the normal and high CNS groups (P = 0.021, and P = 0.048 respectively). Also, the neck circumference to height ratio was greater in the obese group compared to the normal group, but not the high CNS group (P = 0.04; Table 2). Further, the CNS was not associated with the girth to height ratio or the neck to height ratio (P = 0.4; P = 0.08 respectively; S1A and S1C Fig), but was associated with the BW to height ratio (CNS of 0 > CNS of 3, P = 0.035; S1E Fig). The BCS was not associated with any morphometric ratio (girth:height ratio, P = 0.08; neck:height ratio, P = 0.098; and BW:height ratio, P = 0.11; S1B, S1D and S1F Fig).

**Table 2:** The median [IQR] for morphometric measurements taken in 26 mixed-breed ponies.

Morphometry	Unit	Normal	Obese	High CNS	P value
Height^	cm	139 [46]	104 [34.5]	103.5 [21]	0.135
Bodyweight	kg	280 [225]	195 [172]	175 [49]	0.296
BW:Height^	kg/cm	2.2 [1]	2.1 [1]	1.7 [0.5]	0.202
BCS	/5	5.5 [2]^a^	8 [1]^b^	7 [1]^c^	<0.05
CNS	/9	1 [2]^a^	2 [2.5]^ab^	3 [0]^b^	<0.001
Girth circumference^	cm	165 [42.6]	140.5 [33]	130 [20.6]	0.132
Neck circumference^	cm	85 [19.9]	86.5 [14]	77.25 [15.1]	0.28
Girth:Height^	cm/cm	1.218 [0.09]^a^	1.445 [0.2]^b^	1.245 [0.1]^a^	0.021
Neck:Height^	cm/cm	0.641 [0.13]^b^	0.832 [0.21]^a^	0.729 [0.04]^ab^	0.044

**Key:** Values with different superscripts within the same row differ from one another (P < 0.05).

^three ponies are not included in these analyses due to missing data (Normal, n = 8; Obese, n = 5; High CNS, n = 10)

BCS; body condition score, BW; bodyweight, CNS; cresty neck score

### Animal groups and ID

**Fig 2:**
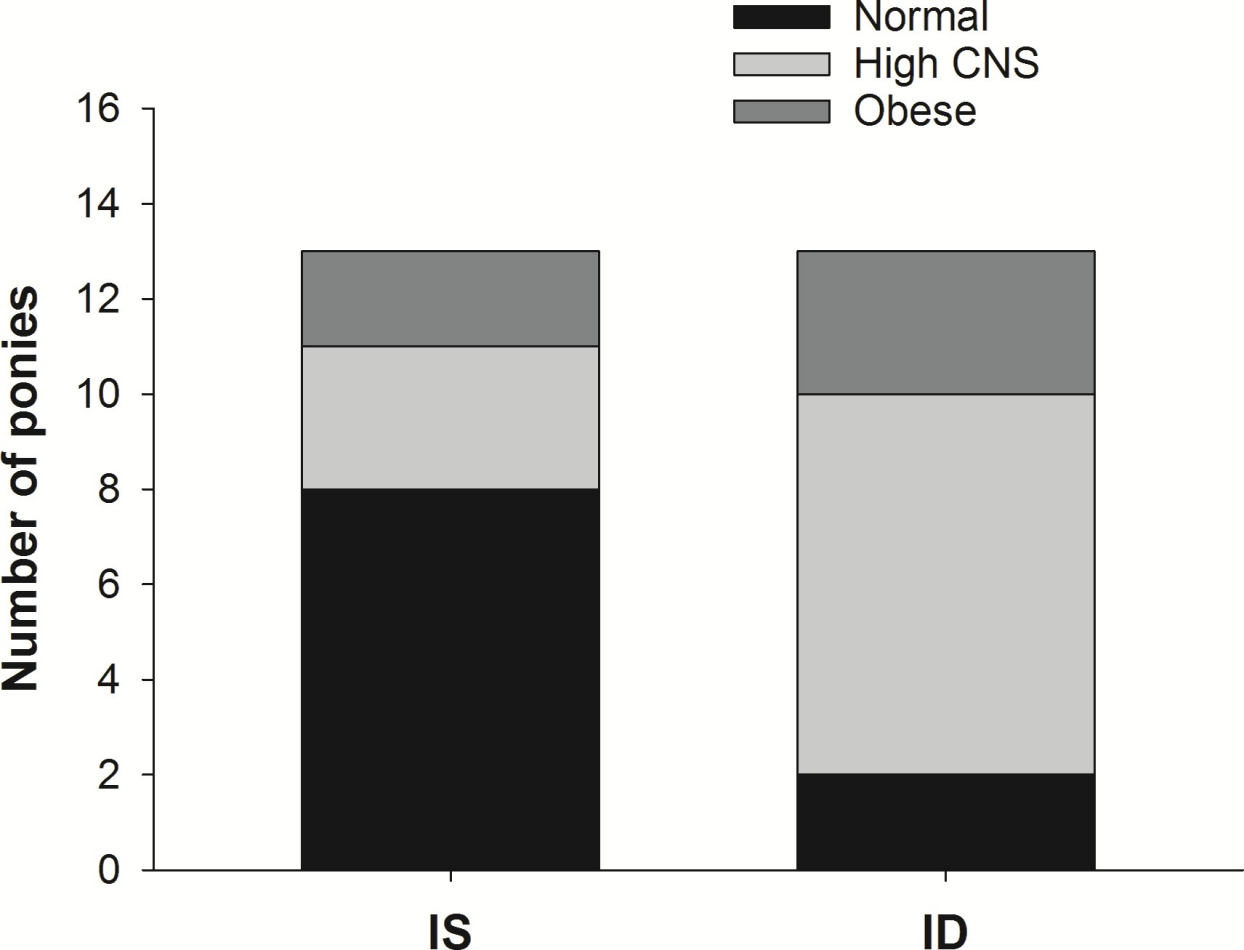
The relationship between generalised obesity, cresty neck score (CNS) and insulin status. Twenty-six ponies were diagnosed as having insulin dysregulation (ID) or not (IS) based on an oral glucose test, and were grouped according to body type. The diagnostic threshold for post-prandial insulin concentration was ≥80 μIU/mL.

Of the entire cohort, 13 ponies were classified as having ID. Of these 13 ponies two had ideal body condition, three were obese and eight had a high CNS (Fig 2). Thus, a cresty neck on an otherwise normal body conditioned pony appears to be a strong predictor of ID. Further, of the 13 ponies without ID eight were in ideal body condition, two were obese and three had a high CNS.

### Logistic regression model

Ponies with a CNS ≥3/5 had five times greater odds (95% CI 1–31.5; P = 0.05) of also being ID than ponies with a CNS ≤2/5. A BCS of ≥8/9 was not found to influence the odds (95% CI 0.26–20.5; P = 0.5) of being insulin-dysregulated.

Further modelling was performed to elucidate the relationship between the BCS, CNS and ID. This revealed that ponies with a high CNS and low BCS had 10.7 times greater odds of being insulin-dysregulated than those with a low BCS and low CNS (Table 3), whereas a high BCS (with or without a high CNS) did not significantly increase the odds of the pony also being insulin-dysregulated.

**Table 3:** The odds of a pony being insulin-dysregulated or not when not obese/low CNS, obese/low CNS, not obese/high CNS, and obese/high CNS.

Group	Exp(β)	95% CI	P value
BCS ≤7/9 and CNS ≤2/5	Referent	-	-
BCS ≥8/9 and CNS ≤2/5	8	0.46–139.3	0.15
BCS ≤7/9 and CNS ≥3/5	10.7	1.38–82	0.02
BCS ≥8/9 and CNS ≥3/5	4	0.17–95.8	0.39

**Key:** BCS, body condition score; CNS, cresty neck score; CI, confidence interval

While both models found BCS to not to be significant when calculating the odds of being insulin-dysregulated, it is still important to note that 3/5 ponies in the obese group were insulin-dysregulated.

### Glucose and insulin

The basal (fasted) blood glucose concentrations did not differ among groups (Table 4). However, blood glucose concentration was positively associated with the BW to height ratio (r = 0.45, P = 0.03). The basal serum insulin concentrations also did not differ among groups. For post-prandial insulin, the high CNS group had higher responses to the OGT compared to normal ponies, and there was an increase in the insulin response in ponies with a CNS score of 3 compared to ponies with a CNS score of 0 and 1 (P = 0.035, and P = 0.025 respectively; Table 4). The post-prandial insulin responses of the obese ponies were not different to either the normal or high CNS group’s responses (Table 4).

**Table 4:** The median [IQR] for blood, serum and plasma hormone measurements in 26 mixed breed ponies.

Hormone parameter	Unit	Normal	Obese	High CNS	P value
Post-prandial Insulin	μIU/mL	41.3 [50.5]^a^	190 [267]^ab^	280 [366]^b^	0.006
Basal Glucose	mM	4.8 [0.7]	4.8 [0.4]	4.8 [0.5]	0.591
Basal Insulin	μIU/mL	2 [6.6]	2.6 [17.8]	5.4 [19.4]	0.168
Basal Adiponectin	ng/mL HE	2.4 [4.5]	3.4 [5.3]	0.62 [1.1]	0.019*
Basal Triglycerides^	mg/dL	32.7 [23.4]^a^	51.3 [98.2]^ab^	48.7 [39.2]^b^	0.04
Basal Leptin	ng/mL HE	9.7 [6.4]^a^	24.4 [29.4]^b^	12.6 [13.5]^ab^	0.03

**Key:** Values with different superscripts within the same row differ (P < 0.05).

*Post-hoc testing (Dunn’s test of pairwise comparisons) identified no significant differences. Normal vs High CNS P = 0.06; Obese vs High CNS P = 0.05.

^three ponies are not included in these analysis due to missing data (Normal, n = 8; Obese, n = 5; High CNS, n = 10), HE; human equivalent

### Triglycerides and adipokines

**Fig 3:**
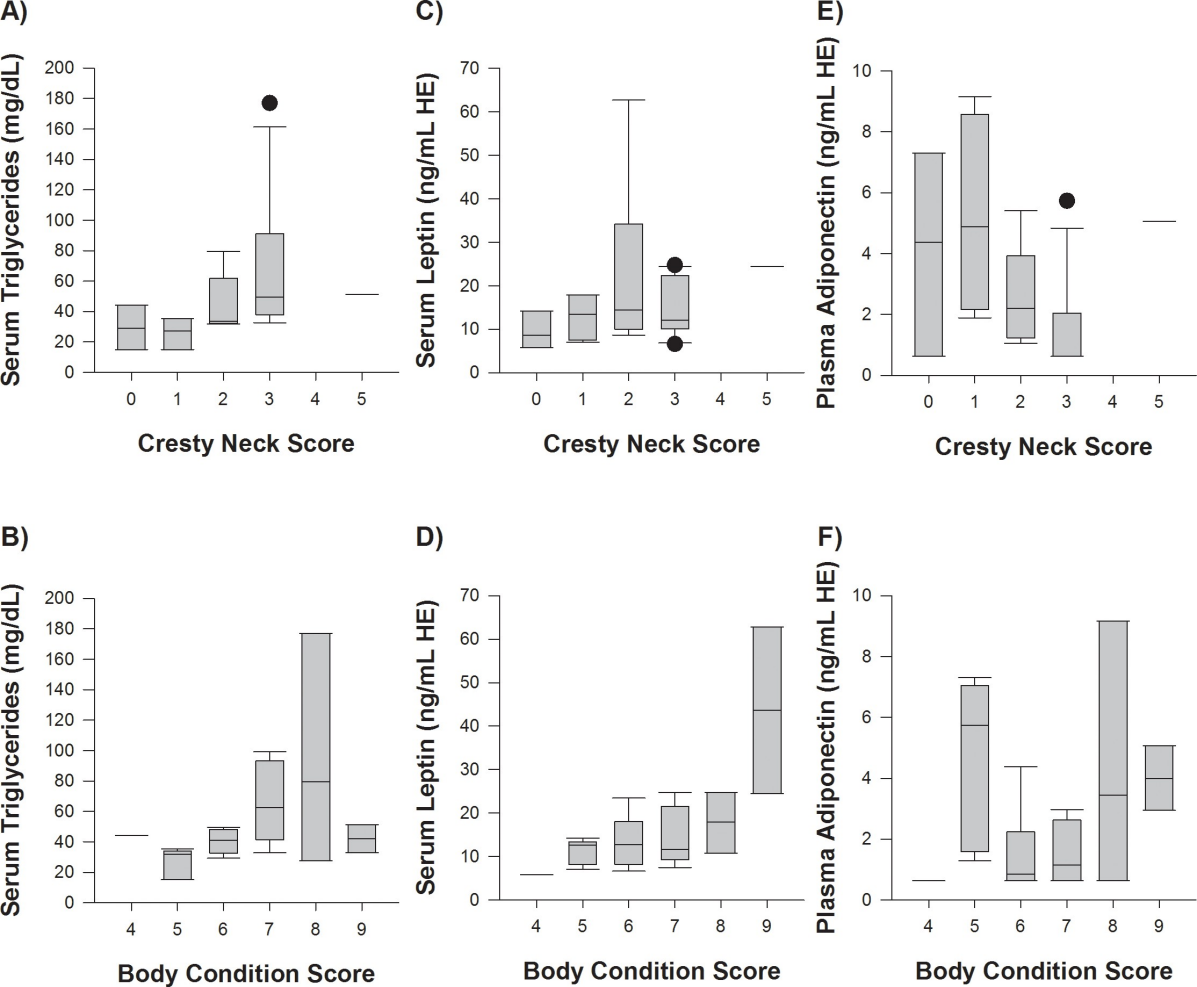
Adipokines, triglycerides and body condition. The association between serum triglycerides (A and B), serum leptin (C and D), and plasma adiponectin (E and F) with the cresty neck score (CNS) and the body condition score (BCS) in 26 ponies.

The high CNS group had higher basal serum triglyceride concentrations compared to normal ponies, but were not different to obese ponies (Table 4). The serum triglyceride concentration was marginally associated with CNS (P = 0.05; Fig 3A). However, serum triglyceride did not differ between the scores for BCS (P = 0.11; Fig 3B).

The basal leptin concentrations were greater in obese ponies compared to normal ponies (Table 4). Furthermore, the high CNS group did not have different leptin concentrations to the normal or obese groups (Table 4). There were no associations between leptin concentrations and the BCS and CNS categories (Fig 3C and 3D).

One pony within the high CNS group was excluded from the basal adiponectin analyses as it was identified as an outlier within the cohort (5.7 μg/mL). Basal adiponectin concentrations were found to be different among groups (Table 4). After post-hoc testing (Dunn’s test), the pairwise comparison demonstrated that the adiponectin concentrations of the normal group were slightly higher (P = 0.05) than the high CNS group. However, the obese and high CNS groups were not different from each other (P = 0.06). Overall, basal adiponectin concentrations were positively associated with the BW to height ratio (r = 0.59, P = 0.003; S2A Fig) and negatively associated with post-prandial insulin concentrations and basal triglycerides (r = -0.5, P = 0.009 and r = -0.46, P = 0.03 respectively; S2B and S2C Fig). Basal adiponectin did not differ between the categories of either the CNS or the BCS (P = 0.09 and P = 0.14 respectively, Fig 3E and 3F).

## Discussion

Within this cohort, ponies with a CNS of 3 or greater were five times more likely to be insulin-dysregulated than those with a CNS below 3, irrespective of their BCS. Additionally, no objective body measurements could be substituted for the CNS in terms of their predictive power. This finding agrees with current thinking that measures of regional adiposity have strong associations with ID, but the role of obesity *per se* in identifying EMS is less important [4, 20]. Studies have examined various morphometric measures in relation to EMS [20, 32, 33]. However, to the authors’ knowledge, the current study is the first to compare ponies displaying a high CNS alone with obese and normal ponies with respect to ID.

The CNS limit of ≥ 3 suggested in the current study as being a threshold for increased risk of ID is supported by Giles et al. [6] who found that ponies with a CNS of ≥ 3 had increased odds of developing laminitis. In other species, regional depots of adiposity (such as visceral adipose tissue in humans) are more closely related to metabolic dysfunction than other fat stores (e.g. subcutaneous fat) [23, 34]. A similar phenomenon may be occurring in the horse, with some work suggesting that nuchal fat and tail head fat may have different endocrine characteristics, compared to visceral fat [35].

Although this study suggests that nuchal adiposity may be of greater interest in relation to ID than generalised obesity, obese ponies may still be insulin-dysregulated. In this study two obese ponies were insulin-dysregulated, while two were not, therefore a greater sample size of obese ponies with a range of cresty neck scores would be required to further elucidate the role of generalised obesity in EMS. The association of generalised obesity with insulin regulation has been studied, with results indicating that an increase in both the BCS and CNS do not necessarily result in a decrease in insulin regulation; instead insulin sensitivity is influenced by diet and breed [25]. Another study reported that the morphometric measurements of girth to height ratio, neck circumference to height ratio, and BCS all correlated well with basal insulin and glucose measures (and ratios) in a cohort of Arabian horses (CNS not measured) [13], demonstrating the risk of increased adiposity to metabolic health.

The scoring systems of BCS and CNS are useful for experienced assessors and cannot be used interchangeably. However, these scores are subjective, and therefore can be imperfect [9]. In addition, previous research has shown that nuchal fat is a poor predictor of total body fat [9], that not all obese animals have a high CNS [26], and as seen here, that ponies can have a high CNS without a high BCS. Thus, an objective measure to assess obesity and adiposity of the nuchal crest would be useful, especially for inexperienced assessors [12, 36]. The use of a BW to height ratio in the current study distinguished ponies with no visible appearance of a crest from those with an enlarged and thickened crest. However, the neck to height ratio was unable to distinguish those with an increased CNS. The subcutaneous fat depth at the base of the neck was identified as a potential objective measure of EMS in Andalusian horses [37]. However, extensive breed-specific validation is necessary prior to the generalised use of this measure.

The human HMW adiponectin ELISA used in this study performed reasonably well with equine plasma, although it did not perform as well as the company’s previous iteration of the kit [29]. The ELISA was not suitable for measuring lower concentrations, and an increase in the LOD is recommended (to 0.625μg/mL). Other methods of measuring adiponectin have been studied. However, none have validated well for use with equine plasma [30]. Due to HMW adiponectin being of interest to EMS and laminitis, it may be useful for a more accurate quantification method to be devised, or for an equine specific kit to be manufactured.

Adiponectin is secreted by white adipose tissue and has an insulin-sensitising effect on tissues, with the HMW multimer best correlated with insulin sensitivity in humans [38]. In the current study, HMW adiponectin concentrations were lower in ponies with cresty necks than those of a normal body condition status. A larger sample size would be necessary to elucidate whether a difference between ponies with cresty necks and generalised obesity exists (P value of 0.06). Previous reports show that HMW adiponectin concentrations decrease as the body condition score increases [29]. However, the horses in that study also had CNS >3 and relatively high non-fasted basal insulin concentrations. The current results align with the hypotheses that: 1) HMW adiponectin is decreased in horses with abnormal insulin regulation [25, 39]; and 2) that HMW adiponectin is not altered in horses with increased adiposity [20]. Adiponectin continues to be of interest in other models of metabolic dysfunction where low adiponectin concentrations have been associated with increases in insulin resistance and visceral fat in humans [22] and cats [40, 41]. HMW adiponectin may be a suitable hormone for the identification of ID in horses, hence the importance of developing a reliable, equine-specific assay.

Hyperleptinemia has previously been described as a component of EMS [18, 42]. Serum leptin concentrations in ponies increased as BCS increased [20, 43] and were higher in ponies with ID, with these ponies also having greater BCS and CNS than the control groups [18, 44]. However, these studies did not discriminate between obese ponies, and ponies with ‘normal’ BW with cresty necks. Interestingly, in a cohort of Andalusian horses, basal insulin and leptin concentrations increased with subcutaneous fat depth at the base of the neck [37]. However, leptin was more strongly correlated with BCS in these horses, indicating that subcutaneous fat depth at the base of the neck may be related to BCS. Hyperleptinemia does reduce tissue insulin sensitivity [45], with the results presented here supporting that leptin concentrations are greater in obese ponies, but are not greater in ponies with a high CNS alone. This raises the question of what the role of leptin is in EMS, particularly in animals with post-prandial hyperinsulinemia.

Triglycerides in the blood increase as fat is mobilised to meet energy requirements and after a meal. Basal triglycerides were higher in horses with ID [18] and in obese horses [43]. Additionally, triglycerides increased over a period of diet restriction in obese ponies [46], demonstrating that fat can be mobilised when on a decreasing plane of nutrition. In the current study, ponies displaying a high CNS, and some obese ponies, had relatively high basal triglyceride concentrations, which could reflect a change in energy requirements in these ponies after fasting overnight. Additionally, a state of hyperinsulinemia causes a reduction in the abundance of lipid and fatty acid transporters in adipose tissue, while increasing the abundance of lipid transporters in skeletal muscle in horses [47], which may contribute to an increase in circulating triglycerides. In humans, serum triglycerides have been positively associated with visceral fat, but not subcutaneous fat [48]. This may help to explain our finding of a difference in basal triglycerides in ponies with a high CNS compared to normal ponies, but that there was no difference between obese and normal ponies.

### Limitations

A limitation of this study was that the normal group was classified as a BCS≤7 and CNS≤2, where a BCS of 7 is considered to be overweight in many studies [7, 25, 49, 50]. However, a BCS of 7 has also been considered as a separate sub-group (labelled “over-conditioned”), rather than falling within the obese category [51]. This classification was used to consolidate the two scores and highlight when ponies appeared to be storing fat preferentially along the nuchal ligament that had not yet been masked by extensive general adiposity. An additional consideration is that ponies in Eastern Australia generally exhibit higher adiposity than horses [26] and therefore require some adjustment of the scale when compared to the wider equine population. Further, this study exclusively used ponies so extrapolation of these data to horses requires some caution. Lastly as addressed above, having only five ponies in the obese group was a potential limitation of this study.

## Conclusion

Adiposity along the nuchal ligament (as measured by the CNS) is a useful tool for identifying the risk of ID (or hyperinsulinemia) in ponies. While generalised obesity should always be managed, it was not a particularly strong a predictor of ID in this population. Basal HMW adiponectin might be a useful marker for hyperinsulinemia (or ID), and further work to clarify the role of adiponectin is warranted and improved assays for use with equine plasma are needed.

## Supporting information

S1 Fig Association between body scores and morphometric ratios in ponies. The association between the cresty neck score and the body condition score with the morphometric ratios of girth circumference to height, neck circumference to height and body weight (BW) to height.
(TIF)

S2 Fig Association between adiponectin and measures of morphometry and insulin dysregulation in ponies. The basal plasma adiponectin was positively associated with the body weight (BW) to height ratio (A), and negatively associated with the post-prandial serum insulin concentration (B) and the basal serum triglyceride concentration (C).
(TIF)
