# Supplementary figures and images for: The cresty neck score is an independent predictor of insulin dysregulation in ponies
Source: PLoS One. 2019 Jul 24;14(7):e0220203. doi: 10.1371/journal.pone.0220203 (PMC6655749; doi:10.1371/journal.pone.0220203)

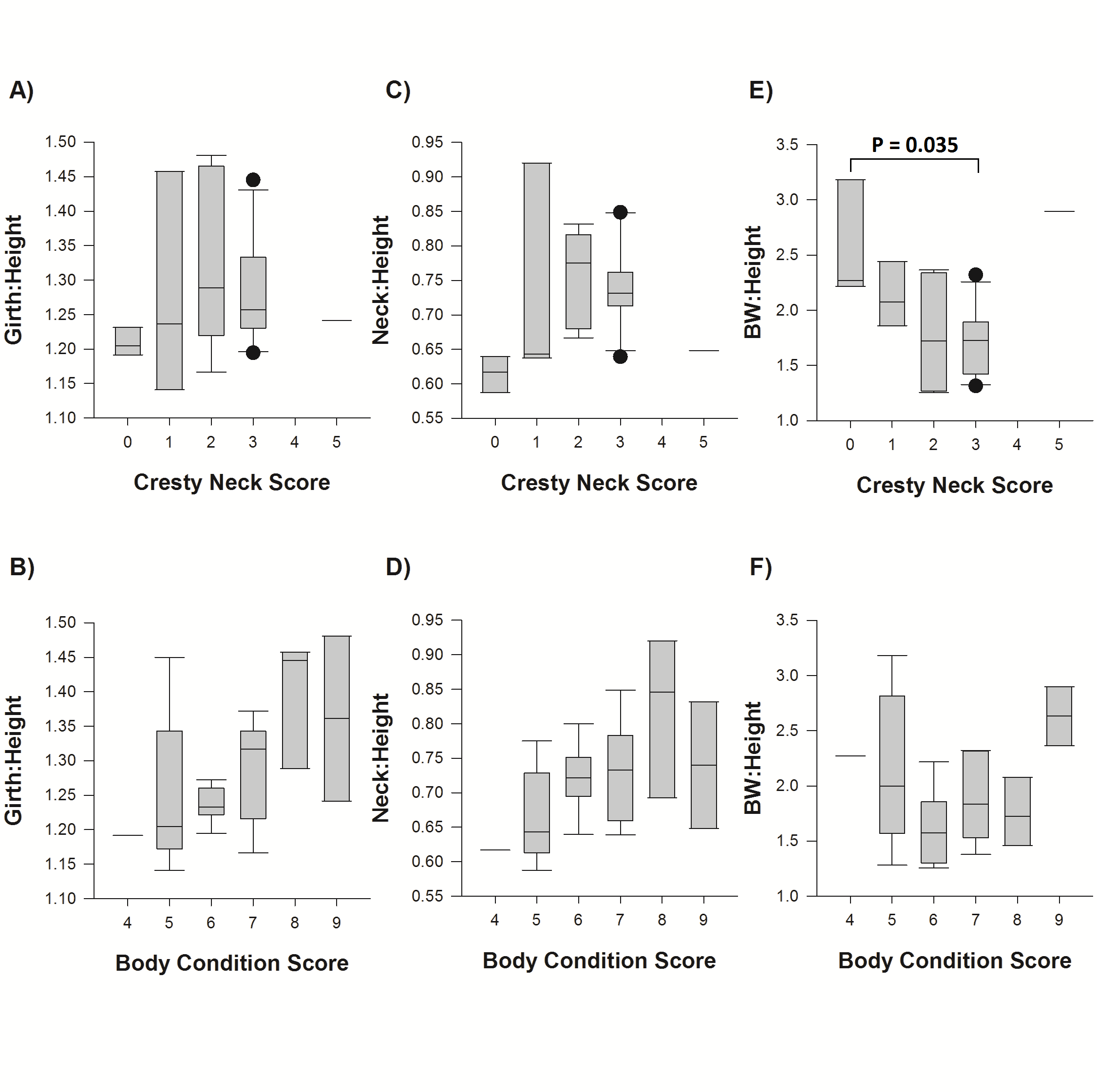

Supplement: S1 Fig — The association between the cresty neck score and the body condition score with the morphometric ratios of girth circumference to height, neck circumference to height and body weight (BW) to height. (TIF) [file pone.0220203.s001.TIF]

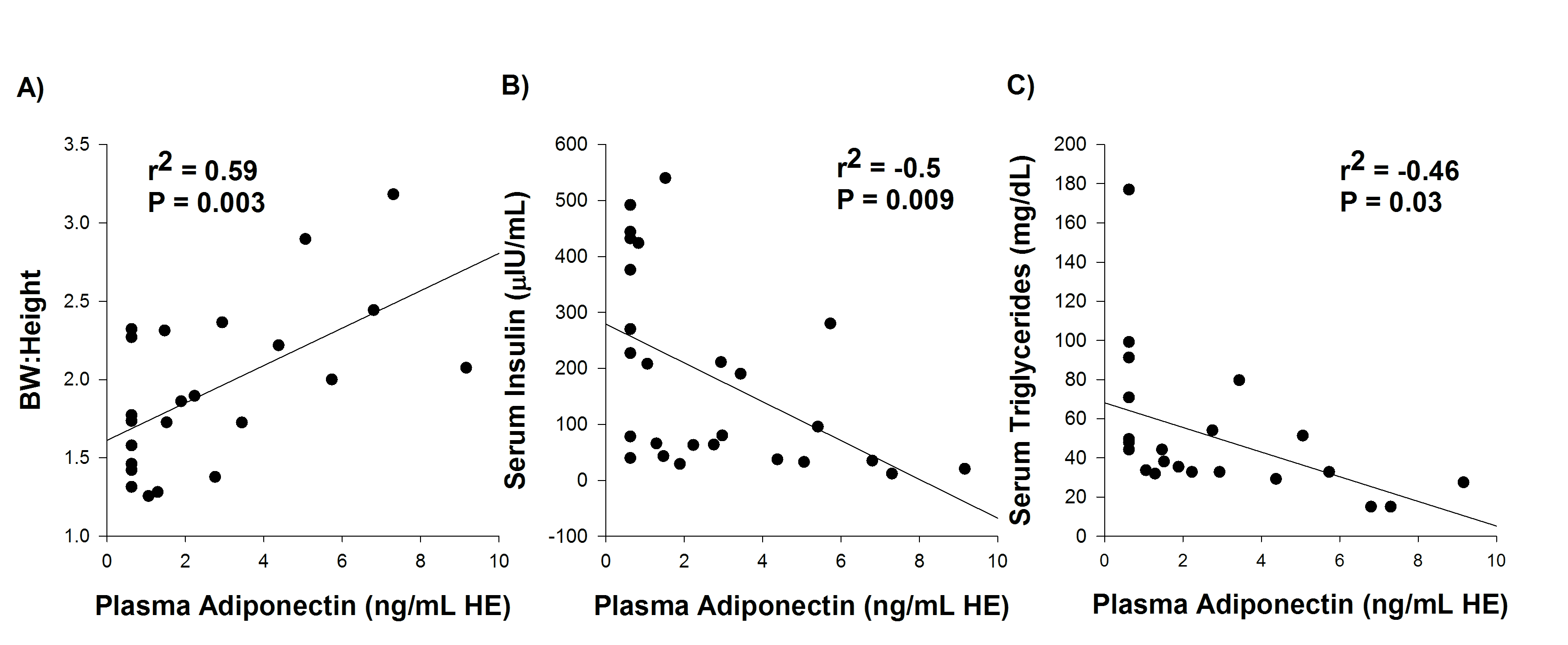

Supplement: S2 Fig — The basal plasma adiponectin was positively associated with the body weight (BW) to height ratio (A), and negatively associated with the post-prandial serum insulin concentration (B) and the basal serum triglyceride concentration (C). (TIF) [file pone.0220203.s002.TIF]
